# Allele frequency misspecification: effect on power and Type I error of model-dependent linkage analysis of quantitative traits under random ascertainment

**DOI:** 10.1186/1471-2156-7-21

**Published:** 2006-04-20

**Authors:** Diptasri M Mandal, Alexa JM Sorant, Larry D Atwood, Alexander F Wilson, Joan E Bailey-Wilson

**Affiliations:** 1 Department of Genetics Louisiana State University Health Sciences Center 533 Bolivar Street, CSRB 6-16 New Orleans, LA 70112 USA; 2 National Human Genome Research Institute, NIH, Baltimore, MD, USA; 3 Department of Neurology, Boston University School of Medicine, Boston, MA, USA

## Abstract

**Background:**

Studies of model-based linkage analysis show that trait or marker model misspecification leads to decreasing power or increasing Type I error rate. An increase in Type I error rate is seen when marker related parameters (e.g., allele frequencies) are misspecified and ascertainment is through the trait, but lod-score methods are expected to be robust when ascertainment is random (as is often the case in linkage studies of quantitative traits). In previous studies, the power of lod-score linkage analysis using the "correct" generating model for the trait was found to increase when the marker allele frequencies were misspecified and parental data were missing. An investigation of Type I error rates, conducted in the absence of parental genotype data and with misspecification of marker allele frequencies, showed that an inflation in Type I error rate was the cause of at least part of this apparent increased power. To investigate whether the observed inflation in Type I error rate in model-based LOD score linkage was due to sampling variation, the trait model was estimated from each sample using REGCHUNT, an automated segregation analysis program used to fit models by maximum likelihood using many different sets of initial parameter estimates.

**Results:**

The Type I error rates observed using the trait models generated by REGCHUNT were usually closer to the nominal levels than those obtained when assuming the generating trait model.

**Conclusion:**

This suggests that the observed inflation of Type I error upon misspecification of marker allele frequencies is at least partially due to sampling variation. Thus, with missing parental genotype data, lod-score linkage is not as robust to misspecification of marker allele frequencies as has been commonly thought.

## Background

In model-based linkage analysis, one assumes that the causative mechanisms of both the trait and marker phenotypes are known without error, including the number of loci involved, the mode of inheritance and allele frequencies. With the advancement of genetic research in complex traits, we are dealing more with uncertain mode of inheritance and unknown parameters (allele frequency and degree of dominance) which ought to be known to apply model-dependent linkage analysis appropriately. Several studies have been reported in the literature about the implications of misspecifying the underlying genetic model of the trait in linkage analysis. Clerget-Darpoux et al. [1] quantified the effects of using wrong allele frequencies, penetrance and degree of dominance on the linkage test and on the recombination fraction estimate under the assu mption of a single locus model in lod-score linkage analysis. Goldin and Weeks [2] reported that a biased estimate of the recombination fraction is introduced in lod-score analysis by reducing the power to detect an existing linkage when underlying disease model parameters are incorrectly specified in the case of a common dichotomous trait. It is important to mention that the accurate estimation of marker allele frequencies is essential in the linkage analysis when reconstruction of the genotypes of key family members is needed due to the unavailability of biological samples (e.g., in the case of diseases with late age of onset, such as lung cancer, when parental data often are not available). So far, a few studies have been presented in the literature about the effect of using incorrect allele frequencies in linkage analysis. Ott [3] and Freimer et al. [4] reported an increase of the false positive rate in linkage when incorrect marker allele frequencies are assumed in the analyses and selection takes place through trait phenotypes. Misspecification of the assumed marker model also leads to an elevated Type I error rate when linkage analysis is performed on pedigrees ascertained with respect to the trait phenotypes [5]. Asymptotically, bias is NOT introduced in the linkage analysis if there is a misspecification of the trait related parameters and the ascertainment is through the trait phenotypes or if there is a misspecification of the marker related parameters and the ascertainment is through the marker phenotypes [6].

In previous studies, [7,8] the power of lod-score linkage analysis using the correct model for the trait appeared to increase when either some or all of the parental marker information was missing and the marker allele frequencies were misspecified in the analysis. An investigation of Type I error rates was then conducted in the absence of parental data and with misspecification of marker allele frequencies. This showed that an inflation in Type I error rate was the cause of at least part of this apparent increased power [8,9]. The objective of the current study is to investigate, using a different analytic strategy, whether the observed inflation in Type I error rate in model-based lod-score linkage is due to sampling variation.

## Methods

### Simulation of data

Computer simulations generating a quantitative trait and marker data in nuclear families were performed using G.A.S.P. V3.3 [10]. The trait simulated was due to an additive major locus with two equifrequent alleles. The heritability of the trait due to the single trait locus was 90%, with the remaining phenotypic variation (10%) due to a residual environmental component. Data were simulated for highly polymorphic five-allele marker loci linked to the trait locus with recombination fractions of 0.0 and 0.01, as well as an unlinked marker, and with varying combinations of allele frequencies. The simulated marker allele frequencies were 0.5, 0.3, 0.2, 0.1, 0.05, 0.01 and 0.001 for the "first" allele, with the other four alleles being equally frequent. For each marker model, 10,000 samples of 300 nuclear families with sibship size of two (300 independent sibpairs) were simulated. Marker data for all parents were suppressed (missing), in order to provide the situation in which marker allele frequencies are used in lod-score linkage analysis. These samples were then analyzed in two different ways, as described below.

### Analyses

#### LOD-score linkage analysis assuming the "true" generating model for the trait

Lod-score tests of linkage between the trait and both linked and unlinked marker loci were performed using LODLINK [11] on each of 10,000 samples for each marker model. LODLINK is a model-based linkage analysis program, where it is assumed that one can fully describe the mode of inheritance of the disease gene, i.e., its allele frequency and the penetrances for each disease locus genotype are known. The parameter values used in the simulation to generate the data were used as the assumed trait model in these linkage analyses. Analyses were repeated for different assumptions about marker allele frequencies. A range of values between 0.001 and 0.6 were used for the "first" allele, and the remaining alleles were assumed equally frequent (Table 1).

**Table 1:** Allele frequencies (first, rest) in a five-allele marker used in the study. The frequency of the most common marker allele is underlined. All combinations of true and assumed allele frequencies were tested.

True allele frequencies	0.001, 0.24975	0.01, 0.2475	0.05, 0.2375	0.1, 0.225	0.2, 0.2	0.3, 0.175		0.5, 0.125	
Assumed allele frequencies	0.001, 0.24975	0.01, 0.2475	0.05, 0.2375	0.1, 0.225	0.2, 0.2	0.3, 0.175	0.4, 0.15	0.5, 0.125	0.6, 0.1

#### LOD-score linkage analysis assuming a trait model estimated from the data

The same simulated data were reanalyzed using LODLINK as described above except that instead of assuming the "true" simulation model, parameters estimated by segregation analysis of the trait data were assumed for the trait model. The segregation analysis was performed on each replicate sample using REGCHUNT [12], an automated quantitative trait segregation analysis program that fits models using the method of maximum likelihood using many different sets of initial estimates. REGCHUNT incorporates the functionality of REGC [11] running multiple times. This program generates the initial values randomly from a uniform distribution. It discards invalid initial estimates and aborts maximizations in which an allele frequency goes to a bound. For this study, 100 sets of initial estimates in a Mendelian three-distribution model were used for model fitting; and the best fitting model obtained from each sample was used in the subsequent linkage analysis of that sample.

### Power and Type I error rates

For both methods of analysis, power was determined as the proportion of 10,000 samples in which analysis of a marker linked to the trait produced maximum LOD scores equal to or exceeding thresholds corresponding to nominal significance levels of 0.01, 0.001 and 0.0001. Type I error rate was determined in a similar manner based on analysis of the unlinked marker, using nominal significance levels of 0.01 and 0.001, since 10,000 replications are not adequate to accurately estimate Type I error at the 0.0001 level. Note that commonly used lod-score thresholds of 1.0, 2.0 and 3.0 correspond to p-values of approximately 0.0159, 0.0012, and 0.0001, respectively [13,14].

## Results

### Observed power to detect linkage using the generating trait model

**Figure 1:**
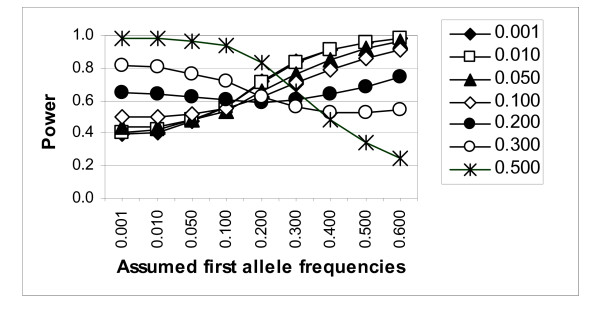
Power of model-based lod-score linkage analysis (for the 0.0001 significance level), assuming the generating model, for a marker at recombination fraction 0.01. Simulated ("true") marker allele frequencies of the "first" allele are indicated by different symbols as presented in the legend.

The power of lod-score linkage analysis of a linked marker (recombination fraction 0.01), using the "correct" generating model for the trait, is presented in Figure 1 for the 0.0001 significance level. The power was found to either increase or decrease in some situations when the marker allele frequencies were misspecified in the analysis. For a significance level of 0.0001, the power increased from 39% to 56% when the "true" marker allele frequencies of 0.001, 0.24975, 0.24975, 0.24975, and 0.24975 were misspecified as 0.1, 0.225, 0.225, 0.225, and 0.225, respectively. A similar trend was observed in the other situations when the frequency of the most common marker allele(s) was underestimated for the analysis (hence, the less common allele(s) overestimated). The power decreased when the frequency of the most common allele(s) was moderately overestimated (e.g., power went down from 55% to 50% at a significance level of 0.0001 when the most common allele frequency of 0.225 was overestimated as 0.24975). The same pattern held true for other significance levels and for the more tightly linked marker, and, as expected, the power was generally greater at the more tightly linked marker (data not shown).

### Observed power to detect linkage using the estimated trait model from REGCHUNT

**Figure 2:**
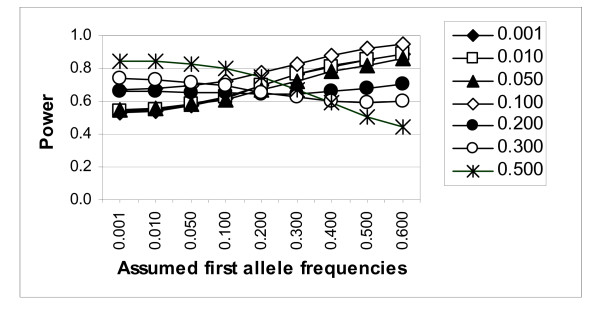
Power of model-based lod-score linkage analysis (for the 0.0001 significance level), using the sample-specific estimated trait model, for a marker at recombination fraction 0.01. Simulated ("true") marker allele frequencies of the "first" allele are indicated by different symbols as presented in the legend.

Where the power using the generating trait model showed an extreme inflation (or deflation) due to the misspecification of marker allele frequencies in the analysis, inflated (or deflated) power to detect linkage in the same situation was also observed using the estimated trait model from REGCHUNT. However, the extent of inflation (or deflation) was less than when using the generating trait model, except in some cases when inflation was limited by the upper bound of 100%. For significance levels 0.01 and 0.001, the power was always lower, compared to analyses assuming the true (generating) trait model (data not shown). However, the reverse was frequently true for significance level 0.0001. For example, when "true" marker allele frequencies of 0.001, 0.24975, 0.24975, 0.24975, and 0.24975 were misspecified as 0.1, 0.225, 0.225, 0.225, and 0.225, respectively, the power increased from 53% to 62%. Results are presented in Figure 2 for the 0.0001 significance level, using the marker at a recombination fraction of 0.01. Similar trends were observed for the tightly linked marker (data not shown).

### Observed false-positive error rates using the generating trait model

**Figure 3:**
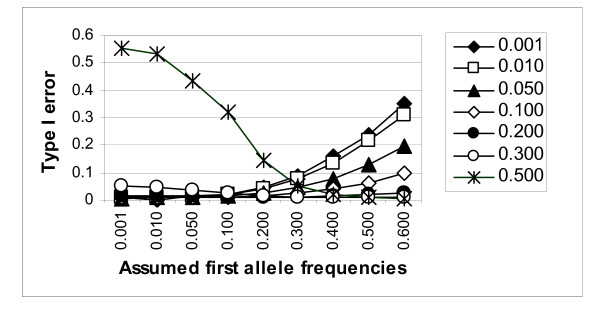
Type I error of model-based lod-score linkage analysis (for the 0.01 significance level), assuming the generating model, for a marker at recombination fraction 0.5. Simulated ("true") allele frequencies for the "first" allele are indicated by different symbols as presented in the legend.

As the power of lod-score linkage analysis using the "correct" generating model for the trait was found often to increase substantially when the marker allele frequencies were misspecified, nominal type I error rates were investigated. Observed Type I error rates based on linkage tests at the unlinked marker are presented in Figure 3 for the 0.01 significance level. A consistent increase in Type I error was detected when underestimates of the most common marker allele(s) and overestimates of the less common allele(s) were used in the analysis. For example, at a nominal significance level of 0.01, the observed Type I error rate was 0.0087 when the true allele frequencies of 0.01, 0.2475, 0.2475, 0.2475, and 0.2475 were used in the analysis but was inflated to 31% when the allele frequencies in the analysis were severely misspecified as 0.6, 0.1, 0.1, 0.1, and 0.1.

When the most common allele(s) were misspecified as even more common in the analysis, the observed Type I error rate tended to decrease slightly, unless the misspecification was relatively large.

### Observed false-positive error rates using the estimated trait model from REGCHUNT

**Figure 4:**
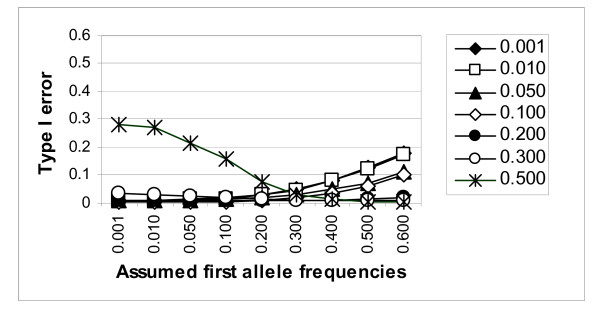
Type I error of model-based lod-score linkage analysis (for the 0.01 significance level), using the sample-specific estimated trait model, for a marker at recombination fraction 0.5. Simulated ("true") allele frequencies for the "first" allele are indicated by different symbols as presented in the legend.

As shown in Figure 4 for the 0.01 significance level, an increase in Type I error was usually observed when the frequency of the most common allele(s) was decreased (misspecified downward from the true value) and that of the less common allele(s) increased in analysis using a sample-specific estimated trait model. This is the same general pattern as that observed when the "true" generating trait model was used in the analyses. However, the observed Type I error rates were usually closer to the nominal levels when the assumed trait models were generated by REGCHUNT than when the generating model was assumed. This was particularly noticeable in cases of extreme misspecification.

## Discussion

When performing lod-score linkage analysis of a randomly sampled quantitative trait with missing parental marker information, the power of lod-score linkage analysis often appears to increase as the marker allele frequencies are misspecified even when the "correct" generating model for the trait is used in the analysis. The effect of misspecification on power is more pronounced when the nominal significance level is more significant. The apparent increase in power appears to be due at least in part to an increase in Type I error rates. A severe increase in the Type I error rate was observed for lod-score linkage analysis in a sample size of 300 independent sibpairs without parental data when marker allele frequencies were misspecified in the analysis by underestimating the most common marker allele. The inflation may be caused by sampling variation resulting in some extreme samples for which the "true" generating model is not a good fit. It is also possible that this inflation in Type I error rate is observed because these sample sizes were not large enough to display asymptotic properties. Different proportions of available parental information have a significant effect on the power of lod-score linkage analysis depending upon the sample sizes [7]. Omission of all parental marker data has a substantial deleterious effect on the power observed in lod-score linkage analysis for small or moderate sample sizes (100 or 300 independent sib pairs). In the case of larger sample sizes (500 independent sib pairs) this effect is observed only at nominal significance levels more extreme than 0.0001. Use of marker information for at least one parent in the analysis reduces the observed Type I error rate to the nominal level or close to the nominal level in many situations. In addition, use of multiple sibpairs from the same sibship has a similar effect by allowing parental genotypes to be imputed and used in the analysis [8].

In an attempt to investigate the effect of misspecification of marker allele frequencies on trait heritability, similar analysis was done on a simulated trait with 50% heritability due to an additive major locus with two equifrequent alleles and the other one due completely to a random environmental effect. LODLINK was used to test for linkage in each of 10,000 replicates of 300 families with sibship size two. When the trait was completely environmentally determined, the Type I error rate of the lod-score linkage analysis was found to be robust to misspecification of marker allele frequencies, even if the allele frequencies were severely misspecified (± 0.8 of true frequencies). However, for the trait with heritability of 50%, the Type I error rate was sometimes as much as 21 times the nominal significance level of 0.001 when the allele frequency of a common allele in a five-allele polymorphic marker was misspecified in the analysis as rare (data not shown). In general, the Type I error rates for the trait with a heritability of 90% were the highest among the three traits studied. Thus, when the parental genotype data are missing, the model-based lod-score test of linkage is robust with respect to Type I error for environmentally determined traits but not for the traits which are largely genetically determined. The results for the trait with heritability of 50% were similar to that of the trait heritability of 90% but less dramatic [9].

In an attempt to resolve the problem of inflated Type I error rates, the model was estimated from each sample using REGCHUNT followed by linkage analysis with LODLINK. The data obtained show that the observed power and Type I error rates of model-based LOD score linkage are often inflated when marker allele frequencies are misspecified, even when using this analytic strategy. However, the Type I error rates observed in this case are usually closer to the nominal levels than those obtained when assuming the generating trait model. This suggests that the observed inflation of Type I error upon misspecification of marker allele frequencies is at least partially due to sampling variation. However, sampling variation is inadequate to completely explain the pattern of inflation of Type I error rates due to marker allele misspecification. The observed Type I error rates are possibly due to not having asymptotic sample sizes. The effect of sibship size is expected to be small relative to the effect of locus heterozygosity on the ability to infer missing parental genotypes from sibship data. If there are two alleles in a marker, no sibship size can determine with certainty the parental mating type. On the other hand, parental mating type can often be determined with certainty (the higher the locus heterozygosity, the more frequently this is possible) in a sibship of size two if there are four or more alleles. From our findings, we have observed that, for two-allele markers, sib-pair sample sizes of 100 and 300 with sibships of size five (i.e., number of families is 10 and 30, respectively) always produce a better power than sib-pair sizes of 100 and 300 with sibships of size two (i.e., number of families is 100 and 300, respectively) when "correct" allele frequencies are used in the analysis. However, as we increased the number of marker alleles to five, the power using 300 sib-pairs with sibships of size five (i.e., the number of families is 30) is not always better than the power achieved using 300 sib-pairs with a sibship size of two (i.e., number of families is 300). However, when only 100 sibpairs are available for study, sibships of size five (i.e., number of families is 10) yield better power than sibships of size two (i.e., number of families is 100) even with a five-allele marker, since power is not optimal for a sample size of only 100 sib-pairs, so the extra power that is obtained by being able to use the multiple sibs in a large sibship to impute the missing parental genotype is important. In general, an increase in the marker heterozygosity leads to more complete inference of parental genotypes [7]. The power with 300 sibpairs is found to be quite high even when we do not have much information to impute the parental genotypes. However, in the case of a disease for which genetic heterogeneity exists, the sample sizes of 300 pairs with 30 families might be more powerful than 300 pairs in 300 families, since power will also not be optimal in this situation. This issue should be explored in future studies.

It is a common practice in simulation studies to use the generating model as the "best" model. The data from the present study suggest that the estimated model generally performs better, with respect to Type I error, than the generating model. Therefore, we need to use caution in interpreting simulation study results obtained using the generating model. These results also suggest that in order to avoid spurious inferences of linkage when performing linkage analysis of randomly ascertained quantitative traits with missing parental data, a very large sample size is needed. Moreover, both the trait model parameters and marker allele frequencies should be estimated from the sample data for randomly ascertained quantitative traits to attempt to reduce the false positive rates.

A thorough investigation was previously conducted on power and Type I error using the model-free Haseman-Elston (H-E) sibpair linkage method [15,16]. Power results were reported on three types of traits with varying portion of random environmental effect (10%, 30% and 50%). The H-E sib-pair linkage method was found to be robust in situations considered with misspecifications of allele frequencies, except for a slight decrease in power when sample size was small and when the marker was not very polymorphic.

## Conclusion

The estimates of marker allele frequencies are irrelevant to linkage analysis when marker genotypes are known for all family members. However, if parental genotypes are not available, accurate estimates of allele frequencies for highly polymorphic DNA marker loci with high heterozygosities are often not available in the underlying study population. If the genotypes at a marker locus are not known and cannot be imputed for a family member, then inaccurate estimates of marker allele frequencies may lead to false positive evidence for linkage if model-based linkage analysis is used. However, if a quantitative trait is well characterized and marker allele frequencies are accurately estimated the type I error rate for the model-based linkage method is not substantially greater than the nominal rate.

In conclusion, the model-based lod-score method of linkage analysis is not robust to misspecification of marker allele frequencies when the families are randomly ascertained and parental marker data are missing, especially when the true trait model is assumed, rather than one estimated from the data. There is some indication, however, that a slight overestimate of the most common marker allele would be safer to use than an underestimate.

## Authors' contributions

DMM participated in the design of the simulation studies, conducted the simulations, interpreted the results and drafted the paper. AJMS participated in the programming, interpretation and helped to draft the paper. LDA gave his expert comments on REGCHUNT. AFW provided his simulation software and helped in the design of the studies. JEBW participated in the design of the simulation studies, interpretation and provided useful suggestions. All authors read and approved the final manuscript.
